# Altered Gray-Matter Volumes Associated With Betel Quid Dependence

**DOI:** 10.3389/fpsyt.2017.00139

**Published:** 2017-08-03

**Authors:** Fulai Yuan, Lingyu Kong, Xueling Zhu, Canhua Jiang, Changyun Fang, Weihua Liao

**Affiliations:** ^1^Health Management Center, Xiangya Hospital, Central South University, Changsha, China; ^2^Department of Radiology, Xiangya Hospital, Central South University, Changsha, China; ^3^School of Humanities and Social Sciences, National University of Defense Technology, Changsha, China; ^4^Department of Oral and Maxillofacial Surgery, Xiangya Hospital, Central South University, Changsha, China

**Keywords:** betel quid dependence, MRI, VBM, dorsolateral prefrontal cortex, ventral medial prefrontal cortex, orbitofrontal cortex

## Abstract

Betel quid (BQ) is one of the most commonly consumed psychoactive substances. It has been suggested to be associated with various health issues, especially oral cancer. Evidence also points to possible decreased cognitive functions after long-term BQ chewing, such as attention and inhibition control. The present study aims to investigate the brain structure basis of BQ chewing in Hunan province of China. Twenty-five BQ chewers and 25 controls were recruited to participate in this study. Voxel-based morphormetry analysis revealed that there were three key regions showing structural differences between BQ chewers and controls, including bilateral dorsolateral prefrontal cortex (DLPFC)/insula, ventral medial prefrontal cortex, and left orbitofrontal cortex. Moreover, the GMV in the DLPFC could potentially predict BQ dependence scores, level of daily BQ chewing, and history of BQ chewing. These results suggested that participants who showed BQ chewing dependence may have deficit in inhibition control and affective decision-making, and the level of deficit was dependent on the level of daily BQ chewing, and history of BQ chewing. Understanding the neurobiology features of BQ chewing would help us develop novel ways to diagnose and prevent BQ dependence.

## Introduction

Betel quid (BQ, also called betel nut or areca nut) is one of most commonly consumed psychoactive substances (1). Consequently, the International Agency for Research on Cancer (2) has categorized BQ as a Group 1 carcinogen. Long-term BQ chewing was associated with various health issues, especially oral cancer and precancerous conditions (3–9). Studies also suggested that there were decreased cognitive functions after long-term BQ chewing, such as attention and inhibition control (10). Recently, neuroimaging studies compared frequent BQ chewers and controls and found that frequent chewers have altered brain structure (11, 12) and resting-state functional connectivity (13–17). Specifically, BQ chewers have decreased functional connectivity in the DMN including ventral medial prefrontal cortex (VMPFC), and orbitofrontal cortex (OFC)/anterior cingulate cortex (ACC) (14).

However, there is little evidence showing there are structural difference between BQ chewers and controls. One pioneer studies examined 33 BQ chewers and 32 controls and found that BQ chewers have less gray-matter volumes (GMV) in the midbrain, right ACC, bilateral dorsolateral prefrontal cortex (DLPFC) and right superior temporal gyrus, and more GMV in the right hippocampal and right precuneus (11). They also found that the GMV in the left DLPFC and right ACC was correlated with the history of BQ chewing (11). Consequently, researchers suggested that BQ chewing should be treated as substance dependence and they can meet the DSM criteria of substance dependence (14, 15). Volkow and colleagues (18–20) argued that addiction is a brain disease, and different addictions showed similar brain alterations, especially for drug-non-dependent frontal regions. Thus, we could also learn from the structural alterations of other addictive behaviors. For example, Ersche and colleagues (21) found that cocaine-dependent individuals had decreased GMV in orbitofrontal, cingulate, insular, temporoparietal, and cerebellar cortex, increased GMV in the basal ganglia. Similarly, Romero and colleagues (22) found that cocaine-dependent subjects presented higher fractional anisotropy values in the anterior cingulate and lower fractional anisotropy values in the anterior-posterior commissure plane.

The present study aimed to investigate the brain structure basis of BQ chewing in Hunan province of China. In this area, people chew dried areca nut while people in other parts of China (especially Hainan and Taiwan) chew fresh areca nuts (11). We also tried to explore if the GMV in certain brain regions could potentially predict the history of BQ chewing. Understanding the neurobiology feature of BQ chewing would help us develop novel ways to diagnose and prevent BQ dependence.

## Materials and Methods

### Participants

Fifty participants in Hunan area were recruited to participate in this study. All research protocols were explained to the participants and approved by the local IRB (Xiangya Hospital of Central South University of Hunan Province, Changsha, China). All participants signed the written consent form before any examinations. BQ chewers (*N* = 25) were recruited from the outpatient department in Xiangya Hospital of Central South University in Changsha, Hunan, China. As reported before (14), these participants meet the DSM-IV criteria for substance use disorders as determined by the Structured Clinical Interview. Controls (*N* = 25) with matched age and education level were recruited from the same area. Participants were excluded if they (i) met criteria for other substance dependence; (ii) have a medical history of any neurological or psychiatric disorder; and (iii) claustrophobia or other disease prevent them from MRI scanning, e.g., any metal implants. All participants have normal or corrected to normal vision. The demographic and clinical characteristics of the participants can be found in Table 1.

**Table 1:** Demographic and clinical characteristics of participants (M ± SD).

	Betel quid (BQ) chewers	Controls	Statistics
Age (years)	29.87 ± 4.71	28.23 ± 5.92	*t*(48) = 1.06, *p* = 0.29
Education (years)	14.39 ± 5.19	17.31 ± 2.87	*t*(48) = −2.47, *p* = 0.017
Betel Quid Dependence Scale	10.87 ± 1.71	–	–
Duration of BQ chewing (years)	12.96 ± 5.05	–	–
Dosage of BQ chewing (g/day)	48.48 ± 17.54	–	–

### Procedures

Participants were asked to come to the Xiangya Hospital to finish the behavior interview and MRI scan. They were asked to read and sign the consent form first and then complete the behavioral interview. The MRI scan took about 20 min to finish. During the scan, images for one high-resolution structural scan were acquired.

### Behavior Interviews

The severity of BQ dependence was assessed by the Betel Quid Dependence Scale (BQDS), which follows DSM-IV criteria (23). It is a 16-item self-report instrument with three factors: physical and psychological urge, increasing dosage, and maladaptive use.

### MRI Protocol

All MRI images were acquired using a Siemens Skyra 3T scanner at Xiangya Hospital. Participants lay in the supine position on the scanner bed. They were instructed to have a rest but keep their head very still during the structural scan. The scan was performed using T1-weighted 3D-Magnetization Prepared Rapid Gradient Echo sequence, covering the whole brain with the following scanning parameters: TR/TE = 1900/2.01 ms, flip angel = 9°, matrix = 256 × 256, number of slices = 176, FOV = 256 mm × 256 mm, and slice thickness = 1 mm.

### VBM Analysis

We used FSL-VBM implemented in FSL (24) to analyze the structural MRI data (25–27). Brains were extracted using BET (28) and segmented into gray matter, white matter and CSF using FAST4 (29). Two steps of registration (linear and non-linear) were performed to register the gray-matter partial volume images to the standard space (MNI152). A study specific template was created by averaging all normalized images. Last, the resulting GMV images were smoothed with an isotropic Gaussian kernel (3 mm). Statistics were performed with FSL non-non-parametric permutation methods (Randomise v2.1) (30). Voxel-wise GLM models were used to test the group difference and correlation between GMV data and BQ chewing. The null distribution at each voxel was constructed using 10,000 random permutations. Multiple comparisons were corrected across the whole brain using the threshold-free cluster enhancement. The mean GMV in each cluster was then extracted for each individual following region of interests analysis standard.

## Results

### Demographics and Clinical Characteristics

The demographic and clinical characteristics for BQ chewers and controls are given in Table 1. The BQ chewers exhibited a mean of 10.87 ± 1.71 BQDS score, a mean duration of BQ chewing of 12.96 ± 5.05 years, and average daily dosage of BQ of 48.48 ± 17.54 g. The two groups did not differ in terms of age (*t*(48) = 1.06, *p* = 0.29), but the control group was educated for longer time (*t*(48) = −2.47, *p* = 0.017). In later analysis, years of education was used as a co-variate in the model.

### Statistical Comparison of VBM Analysis

**Figure 1:**
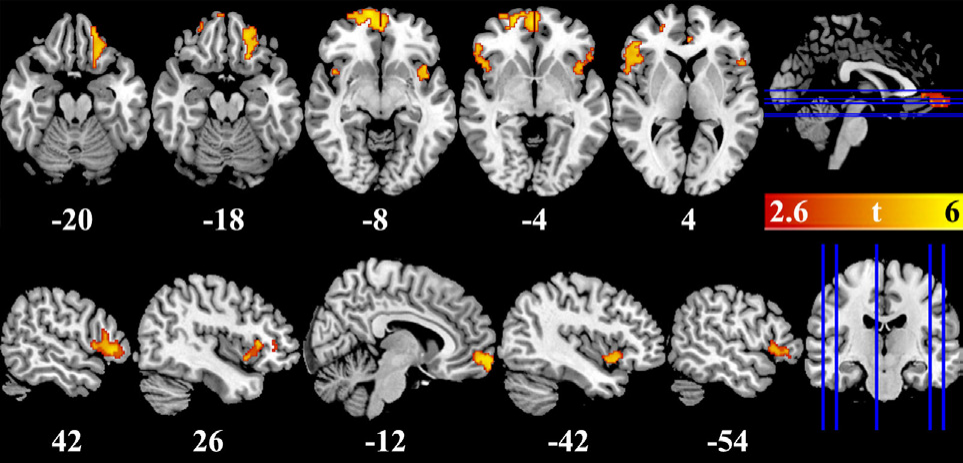
Brain regions showed gray-matter volume difference between betel quid chewers and controls. Figures are displayed in canonical (upper panel) and sagittal (lower panel) view. Numbers below each brain slices were the corresponding *z* (upper panel) or *x* (lower panel) value in the MNI space. Color bar represented the significance of the difference (*t* values).

VBM results showed that, after controlling for education, several brain regions had reduced GMV in BQ chewers compared to controls, including bilateral VMPFC, bilateral DLPFC extending to IFG and insula, and left OFC (Table 2; Figure 1). No brain regions showed higher GMV for BQ chewers than controls.

**Table 2:** Summary of VBM results (Controls > Betel quid chewers).

Brain region		Cluster size	MNI *x*	MNI *y*	MNI *z*	TFCE corrected *p*	*t*
L/R	VMPFC	533	18	58	−4	<0.001	5.46
R	DLPFC/IFG/insula	418	56	28	0	<0.001	4.98
L	OFC	234	−20	28	−16	<0.001	5.04
L	DLPFC/IFG/insula	193	−42	16	−6	<0.001	4.44

*L, left; R, right; MNI, Montreal Neurological Institute, TFCE, threshold-free cluster enhancement; VMPFC, ventromedial prefrontal cortex; DLPFC, dorsolateral prefrontal cortex; IFG, inferior frontal gyrus; OFC, orbitofrontal cortex*.

*The voxel size in VBM analysis is 2 mm × 2 mm × 2 mm*.

Region of interest analysis showed that, in the BQ chewers group, the GMV in left and right DLPFC/insula could predict the BQDS score [left: *r*(25) = 0.536, *p* = 0.006, right: *r*(25) = 0.587, *p* = 0.002], years of BQ chewing [left: *r*(25) = 0.492, *p* = 0.01, right: *r*(25) = 0.615, *p* = 0.001], and daily BQ use [left: *r*(25) = 0.394, *p* = 0.05, right: *r*(25) = 0.413, *p* = 0.04]. It should be noted that, the correlations between DLPFC and daily BQ use were not significant after Bonferroni correction for multiple comparison.

## Discussion

The present study recruited 25 BQ chewers and 25 controls to examine the altered brain structures associated with BQ chewing. Results suggested that there are three key regions presenting structural difference between BQ chewers and controls, including the DLPFC/IFG/insula, VMPFC, and OFC. Especially, the GMV in the DLPFC could potentially predict the BQ dependence score, level of daily BQ chewing, and years of chewing BQ.

These results emphasize that BQ chewers showed similar brain structural alternations as observed in other addictive behaviors. The gray-matter morphology deficit in these regions have been shown to be related to different types of substance and/or behavioral addiction, including cocaine (31–37), heroin (38–41), opiates (42), cannabis (43), nicotine (44, 45), alcohol (46, 47), ketamine (48), MDMA (49), methamphetamine (50), internet (51), and online games (52, 53). For example, Tanabe et al. (54) investigated participants who showed dependence on two or more substances, and results suggested lower GMV in bilateral medial OFC in patients and the neural marker was correlated with decision-making performance in a modified gambling task. It should be noted that, our results were also consistent with a previous reports who suggested similar brain systems’ GMV alternation in BQ chewers (11), especially the DLPFC. Moreover, we also suggested that the GMV reduction in DLPFC was correlated with history of BQ chewing as well as BQ dependence score, which is also consistent with previous reports (11).

These results also implied deficits of two neural systems in BQ chewing, namely the inhibition control system (DLPFC/insula) and affective decision-making system (VMPFC and OFC) (55–59). This idea is also consistent with other types of drug addiction showing that these two systems were altered. For example, drug addiction literature suggested that DLPFC activity reduction in heavy smokers, cocaine users, etc. (20, 60–62). These two systems typically showed hypo-activity in drug addicts, suggesting their impaired ability to resist stimuli that are rewarding in the short term, but lead to negative consequences in the long term (55–58). These two systems, which depend primarily on the functions of the prefrontal cortex, are necessary to control the basic impulses and allow more flexible pursuit of long-term goals (55–58).

Several studies suggest that the cognitive or regulatory control of tempting choices is in part dependent on brain regions that we have hypothesized to be components of the so-called “reflective system” (55–58, 63–67). For example, one study shows that word-level cognitive labels can change the subjective ratings of the affective value of the taste and flavor of a food when the taste or flavor stimulus is identical; this cognitive modulation is expressed in the OFC and ACC (68). This is consistent with the idea that BQ chewers have higher GMV in these two systems, as demonstrated in a previous study (11). In fMRI studies of food consumption, obese men and women had less activation in the left DLPFC in response to a meal than did their lean counterparts (69). While many researchers still clump all mechanisms of decision-making and inhibitory control under one umbrella, the rubric of “Executive Functions,” Bechara et al. (55, 70) have argued that the two are separable neuropsychological mechanisms. More specifically, there is a distinction in functionality between (1) simple inhibitory and impulse control processes mediated by the lateral orbitofrontal and inferior frontal gyrus regions and (2) affective decision-making mediated by VMPFC and OFC, including the frontal pole, which are highly relevant to behavioral control ability and to the decisions individuals make frequently on a daily basis (55). Both inhibitory/impulse control function and affective decision-making are important, specific aspects of higher order executive control functioning (55, 71). Good inhibitory functioning reflects the ability to actively stop a pre-potent behavioral response after it has been triggered. Individuals with deficits or failures in these systems have a tendency to act more impulsively. Adequate affective decision-making reflects an integration of cognitive and affective systems (hence, considered “hot”—emotionally linked—cognition, and the ability to more optimally weigh short-term gains against long-term losses or probable outcomes of an action). Excessive BQ chewing has been known to have short-term “reinforcing effects” but long-term negative consequences should be less likely or problematic for individuals with higher affective decision-making ability.

There are three noteworthy limitations of this study. First, the sample size is relatively small and the participants are all males. Second, the two groups showed significant difference in years of education which could contribute to the GMV difference even though we tried to minimize this effect by adding education as a co-variate. Third, this is a cross-sectional and correlational study, which limits our inference on the causality. Further longitudinal study should be done to replicate and extend the conclusion from this study.

## Ethics Statement

All research protocols were explained to the participants and approved by the local IRB (Xiangya Hospital of Central South University of Hunan Province, Changsha, China.). All participants signed the written consent form before any examinations.

## Author Contributions

FY and WL conceived and designed the experiments. XZ, CJ, and CF conducted the experiments and collected data. XZ and LK analyzed the results. FY, CJ, and CF wrote the main manuscript text. All authors reviewed the manuscript.

## Conflict of Interest Statement

The authors declare that the research was conducted in the absence of any commercial or financial relationships that could be construed as a potential conflict of interest.
